# Editorial: Secondary Effects of Antibiotic Exposure

**DOI:** 10.3389/fmicb.2021.737958

**Published:** 2021-08-24

**Authors:** Amy K. Cain, Laura M. Nolan, Jennifer Cornick, Karl A. Hassan

**Affiliations:** ^1^Department of Molecular Sciences, Macquarie University, Sydney, NSW, Australia; ^2^National Heart and Lung Institute, Imperial College London, London, United Kingdom; ^3^Institute of Infection and Global Health, University of Liverpool, Liverpool, United Kingdom; ^4^School of Environmental and Life Sciences, University of Newcastle, Callaghan, NSW, Australia

**Keywords:** antibiotic, antibiotic resistance, molecular mechanisms, bacterial pathogenesis, host microbe, microscopy, mutagenesis

Most commonly used antibiotics have origins in the chemical wars between diverse microbes competing over millennia in crowded environmental niches, like soil. This enduring need to attack their neighbours has made some microbes ruthless killers armed with the ultimate weapon—antibiotics. Antibiotics target biological functions central to the growth or survival of bacteria with an exquisite specificity that has been honed through countless selective iterations over a very long time.

However, their touted, known mechanisms of action may not be so simple.

The way cells respond to antibiotics are complex and multifactorial, both for the bacterial pathogens and infected hosts. For example, bacteria treated with antibiotics can display a plethora of alterations—physiological, behavioural or regulatory—that are seemingly unrelated to the direct killing mechanisms of the antibiotic. These “off target” secondary impacts of antibiotics can be difficult to explain.

Some secondary impacts might reflect alternative biological functions of the molecules, related to the needs of native producing organisms, such as chemical signalling. Alternatively, they may be collateral effects that do not alter the fitness of the antibiotic producers, or poorly defined downstream consequences of the primary antibiotic targeting mechanism. These illicit effects occur for both fully and semi-synthetic antimicrobials.

This Research Topic in Frontiers in Microbiology focuses on these underexplored secondary effects of antibiotics which impact the physiology, behaviour, and evolution of bacterial, host and other co-localised cells. The secondary effects are frequently subtle, lurking beneath the surface of the primary killing/inhibition action, but become apparent through anecdotal observations, or after prolonged antibiotic use. These effects reveal the secret double lives of antibiotics that have sidestepped significant attention, but whose impacts extend to alterations in bacterial virulence, resistance development and treatment efficacy. The papers in this Research Topic describe targeted and cutting-edge investigations into antibiotic secondary effects and their mechanistic bases. The Research Topic comprises of seven papers, including three reviews and four original research articles that discuss the antibiotic secondary effects that are evident throughout all walks of life. The imperative review papers in the collection discuss the secondary effects of antibiotics on microbial biofilms (Penesyan et al.), antibiotic-induced mutagenesis, as measured using microscopy (Revitt-Mills and Robinson) and bacterial tolerance of antibiotics (Sulaiman and Lam). The outstanding research papers showcase the broad cellular and regulatory effects that the last-line antibiotic tigecycline has beyond inhibiting translation (Li et al.), how antibiotics can trigger increased gene transfer *via* quorum sensing (Shu et al.,), the complex interactions between antimalarials and antibiotics in bacteria (Olateju et al.), and the impact that antibiotics have on animals with brain ischemia (Lee et al.).

Antibiotics impose huge selective pressures on bacteria, and their widespread use is a major anthropogenic driver of bacterial evolution. Two reviews in the collection describe processes related to bacterial adaptation to antibiotics. Revitt-Mills and Robinson address induced-antibiotic mutagenesis, whereby some antibiotics can cause higher than normal mutation rates in bacterial populations, e.g., by modulating processes of DNA synthesis and repair, which may promote population heterogeneity. This heterogeneity theoretically poses greater potential for mutational resistance selection, but the importance of induced-antibiotic mutagenesis in the development of antibiotic resistance has been technically very hard to study because of the range of cellular processes occurring concurrently that could influence resistance development. Revitt-Mills and Robinson describe the use of microscopy to observe directly the molecular mechanisms involved in induced-antibiotic mutagenesis that may then be linked to resistance outcomes.

In the second review describing bacterial adaptation to antibiotics, Sulaiman and Lam explore the phenomenon of antibiotic tolerance, in which a sub-population of bacterial cells can persist during antibiotic exposure in a dormant state. Upon removal of antibiotic selection, these bacterial persister cells may reseed a new bacterial population. Persister cells are not genetically different to their non-persister counterparts. However, various mutations may lead to a higher likelihood of persister cell formation. Sulaiman and Lam catalogue and describe these mutations as identified in laboratory evolution studies.

Bacteria are often found within biofilms, which are surface-associated structures where bacteria, and other microbes, are embedded within a self-produced protective matrix. When bacteria are in biofilms, they are more resistant to many antibiotics as compared to their planktonic counterparts. This results in significant challenges in treating biofilm-associated infections. Given this, many studies have focused on the mechanisms underpinning this increased resistance. In this special edition a very timely review by Penesyan et al., explores how bacterial cells in biofilms respond to sub-inhibitory concentrations of antibiotics and how this impacts upon treatment in healthcare and industrial settings.

Insights into the secondary effects of antibiotics can be gleaned through unbiased genome scale analyses, such as transcriptomics. Tigecycline is an important antibiotic in our arsenal, that is saved for the most difficult and deadly infections, like those caused by the notoriously resistant pathogen *Acinetobacter baumannii*. Its mechanism of action is *via* protein translational inhibition, yet (Li et al.) used transcriptomic approaches to reveal a global network of genes for tigecycline resistance, many of which were out-of-scope for the expected if this antibiotic simply inhibits translation, including changes in toxin-antitoxin systems, peptidoglycan biosynthesis and unrelated antibiotic resistance gene.

Vancomycin is used as the last line of defence against serious infections caused by a range of Gram-positive bacteria. Vancomycin resistant enterococci are widespread and on the WHO priority list of bacteria for which new antibiotics are urgently needed. Pheromone-induced conjugation facilitates rapid dissemination of antibiotic resistance in enterococci populations. Shu et al., demonstrate that transfer of vancomycin resistance in *Enterococcus faecalis* increased dramatically when cells were treated with other antibiotics, namely streptomycin and spectinomycin. The finding that treatment with commonly used antibiotics leads to dissemination of plasmids encoding resistance to different antibiotics is a terrifying and problematic example of the knock-on effects of antibiotic usage.

Microbial co-infections occur relatively frequently, particularly in situations where one pathogen is immunosuppressive, resulting in the need to co-administer different classes of antimicrobials. This raises a very interesting but complicated phenomenon, where the primary or secondary impacts of one antimicrobial may modulate the activity of others. Olateju et al. performed a range of assays to investigate this occurrence between quinoline antimalarials and the beta-lactam antibiotic ampicillin. This research revealed that chloroquine and quinine have limited antibacterial activity alone, but the combination of one of these compound and ampicillin results in at least additive bacterial inhibitory effects. This could be important in the design of treatments for malarial/bacterial co-infections.

Antibiotics are recommended to prevent pathogen infection in patients with brain injury, however in addition to reducing infection risk (Lee et al.) report that the oral administration of antibiotics may lead to further cognitive impairment in patients with brain ischemia. They demonstrate that oral administration of vancomycin and ampicillin to mice with brain ischemia, may lead to neuroinflammation, *via* the increased translocation of LPS into the brain caused by changes in their gut microbiome.

Overall, these reported effects likely represent only the tip of the iceberg of the known secondary roles that antibiotics play within cellular environments. Antibiotics clearly lead a double life that is only now being revealed; in addition to being ruthless and effective killers of bacteria, antibiotics have multifaceted roles in nature that are often overlooked. Future research, especially those employing global molecular approaches, will allow us to fully comprehend the overall consequences of antibiotic treatment on both bacterial pathogens and host cells. Only then can we begin to understand and comprehend the full cellular effects of these complex killing molecules.

## Author Contributions

All authors contributed to the drafting and editing of this editorial.

## Conflict of Interest

The authors declare that the research was conducted in the absence of any commercial or financial relationships that could be construed as a potential conflict of interest.

## Publisher's Note

All claims expressed in this article are solely those of the authors and do not necessarily represent those of their affiliated organizations, or those of the publisher, the editors and the reviewers. Any product that may be evaluated in this article, or claim that may be made by its manufacturer, is not guaranteed or endorsed by the publisher.

